# Long non-coding RNA SNHG3 promotes breast cancer cell proliferation and metastasis by binding to microRNA-154-3p and activating the notch signaling pathway

**DOI:** 10.1186/s12885-020-07275-5

**Published:** 2020-09-03

**Authors:** Hongnan Jiang, Xiaojun Li, Wei Wang, Honglin Dong

**Affiliations:** 1 Department of Breast Surgery, The Second Hospital of Shanxi Medical University, Taiyuan, 030001 Shanxi PR China; 2 Department of Rdaiology, The Second Hospital of Shanxi Medical University, Taiyuan, 030001 Shanxi PR China; 3 Department of Vascular Surgery, The Second Hospital of Shanxi Medical University, No. 382, Wuyi Road, Taiyuan, 030001 Shanxi PR China

**Keywords:** Breast cancer, Long non-coding RNA SNHG3, microRNA-154-3p, Notch signaling pathway, Competing endogenous RNA

## Abstract

**Background:**

Breast cancer (BC) is a malignant tumor that occurs in the epithelial tissue of the breast gland. Long non-coding RNA (lncRNA) small nucleolar RNA host gene 3 (SNHG3) has been found to promote BC cell proliferation and invasion by regulating the microRNA (miR)-101/zinc-finger enhancer binding axis in BC. Herein, the objective of the present study is to evaluate the effect of lncRNA SNHG3 on BC cell proliferation and metastasis with the Notch signaling pathway.

**Methods:**

Differentially expressed lncRNA in BC tissues and normal breast tissues was analyzed. SNHG3 si-RNA-1 and SNHG3 si-RNA-2 were constructed to detect the mechanism of SNHG3 interference in BC cell proliferation, viability, migration and invasion. Then, dual-luciferase reporter gene assay was utilized to verify the binding relation between SNHG3 and miR-154-3p as well as miR-154-3p and Notch2. Moreover, xenograft transplantation was applied to confirm the in vitro experiments.

**Results:**

Highly expressed SNHG3 was observed in BC tissues. The growth of BC cells in vivo and in vitro was evidently repressed after silencing SNHG3. BC cell invasion and migration were inhibited by silencing SNHG3 in vitro. SNHG3 could act as a competing endogenous RNA of miR-154-3p and upregulate the Notch signaling pathway to promote BC cell development. Activation of the Notch signaling pathway can partly reverse the inhibition of cell activity induced by silencing SNHG3.

**Conclusion:**

Our study demonstrated that interfered lncRNA SNHG3 promoted BC cell proliferation and metastasis by activating the Notch signaling pathway. This investigation may offer new insight for BC treatment.

## Background

Breast cancer (BC) is a malignant tumor that occurs in the epithelial tissue of the breast gland and is the most prevalent cancer among the female globally [1]. BC can be triggered by factors like age, menarche history, reproductive patterns, physical activity, breast characteristics and body habitus [2]. Increasing data indicate that incidence and mortality rates in developed countries are declining but growing in developing countries [2]. At present, women give little attention to clinical inspection and examination of BC, thus it is often diagnosed in advanced stage [1]. Surgery, molecular treatment, radiation therapy and chemotherapy are considered as approaches for BC treatment [3]. However, it remains challenging to ascertain an individual basis who would benefit from these treatments while who would be possible to encounter toxicities [4]. In this context, novel therapeutic strategies for BC are in urgent need. Towards this, we undertook a long non-coding RNA (lncRNA)-based approach to understand the underlying mechanism in BC development, in order to develop novel intervention strategies.

LncRNAs are important in disease occurrence and development, and its associations with these diseases contribute to insightful perspectives about the pathogenesis, diagnosis and treatments of diseases [5]. A recent study has suggested that lncRNA regulates gene at transcriptional, post-transcriptional and epigenetic levels to get involved in tumor progression, including BC [6]. Upregulated lncRNA small nucleolar RNA host gene 3 (SNHG3) serves as an oncogene in BC cells [7]. LncRNA SNHG3 serves as a competing endogenous RNA (ceRNA), encouraging the growth of colorextal cancer [8]. Dysregulated miR is observed in many malignancies indicating a tumor suppressive or oncogenic role [9]. It has been reported that miR-154 is a therapeutic target in BC treatment by serving as a tumor inhibitor [10]. Additionally, another study has demonstrated that miR-154-3p is found to be remarkably deregulated in ductal carcinoma in situ, the most common type of non-invasive BC [11]. Notch2 has been found to play an important role in promoting BC cell dormancy and mobilization [12]. Additionally, the Notch signaling pathway is a fundamental mechanism operating in multicellular organisms as well as in most cells, playing a significant role in promoting cell development and differentiation [13, 14]. Notch signaling pathway regulates key target genes’ transcriptional activity and acts as a therapeutic target in treating several cancers, including BC [15]. From all above, it is reasonable to hypothesize that there may be interactions among lncRNA SNHG3, miR-154-3p and Notch2 in BC cell proliferation and metastasis. Thus, we conducted a series of experiments to verify the hypothesis.

## Methods

### Clinical samples

Women with BC were consecutively recruited at the Second Hospital of Shanxi Medical University from January 2015 to January 2018. Before being enrolled in the study, they received routine chest X-ray, mammography and abdominal ultrasonography, but did not receive chemotherapy or radiotherapy. Criteria for exclusion from the study were as follows: inflammatory breast cancer, metastasis, pre-existing treatment or recurrence of the disease, the presence of diseases such as liver disease, arthritis, or other cancers. All patients received radical mastectomy or modified radical mastectomy. Sixty patients diagnosed with BC were recruited in the carcinoma group and sixty patients with benign breast lesions were recruited in the control group. Furthermore, 3 breast cancer and benign breast lesions specimens were collected to perform transcriptome analysis.

### Reverse transcription-quantitative polymerase chain reaction (RT-qPCR)

Trizol (Invitrogen, Carlsbad, CA, USA) was employed to extract total RNA. PrimeScript RT kit (Takara, Bio Inc., Shiga, Japan) was applied to conduct reverse transcription PCR. Quantitative PCR was performed by AceQ qPCR SYBR Green Master Mix kit (Vazyme Biotech Co. Ltd., Nanjing, China) on a LightCycler 480 (Roche, Basel, Switzerland). The primers were synthesized via TransGen Biotech (Shanghai, China). Their sequences are listed in Table 1. All the experiments were performed three times.

**Table 1:** Primers sequence

Sequence	Forward	Reverse	
SNHG3	TTCAAGCGATTCTCGTGCC	AAGATTGTCAAACCCTCCCTGT	
LNC00680	TTCGGTCTCTTCGACGACG	TGCGAACTCTTGGTGTAGGTC	
AC017048.4	CAGCAGAGGAAGACCATGTG	GGCGTTTGGAGTGGTAGAAA	
miR181A2HG	TTGCTGGCGTCTCGGTTAAT	GCCACCACACTGCCATATCT	
AC007461.2	AACGCTT CACGAATTTGCGT	CTCGCTTCGGCAGCACA	
LNC00277	CACGGGGGGCATTTGGAGATTTT	GCCACCACACTGCCATATCT	
GATA3-AS1	CGAGTCGGGTTCTGATCCAC	GGATGCTGCTTTCCACCCAT	
AC017048.3	AGGGGCCTTCCAGATTAAGG	CGAGTCGGGTTCTGATCCAC	
miR-154-3p	GTGGTACTTGAAGATAGGTT	TTGGTACTGAAAAATAGGTC	
Notch1	GTCAACGCCGTAGATGACC	TTGTTAGCCCCGTTCTTCAG	
Notch2	TCCACTTCATACTCACAGTTGA	TGGTTCAGAGAA AACATACA	
Notch3	GGGAA AAAGGCAATAGGC	GGAGGGAGAAGCCAAGTC	
GAPDH	GAAGAGAGAGACCCTCACGCTG	ACTGTGAGGAGGGGAGATTCAGT	

Note: *SNHG3* Small nucleolar RNA host gene 3, *LNC* Long non-coding, *miR* microRNA; *GAPDH* Glyceraldehyde-3-phosphate dehydrogenase

### Cell lines selection

Human BC cell lines MCF-7, MDA-MB-231, HCC1937, BT474, SKBr-3 and breast epithelial cell line MCF10A were purchased from the Experimental Cell Center, Chinese Academy of Sciences (Beijing, China). Subsequently, cells were cultivated in Roswell Park Memorial Institute 1640 medium consisting of 10% fetal bovine serum in a 37 °C incubator with 5% CO_2_ for 48 h and subcultured.

### Small interfere RNA (siRNA)

SNHG3 siRNA-1 and SNHG3 siRNA-2 were synthesized via GenePharma Biotech (Shanghai, China) and transfected using HilyMax kit (Dijindo Laboratories, Kumamoto, Kyushu, Japan) with a firm compliance to its instructions. Afterwards, SNHG3 level was verified with RT-qPCR 48 h later.

### Cell proliferation and viability assays

ZCell proliferation ability was measured as per the requirements of 5-ethynyl-2′-deoxyuridine (EdU) staining [16] and colony formation assay [17]. Cell viability was detected in the light of the instructions of 3-(4, 5-dimethylthiazol-2-yl)-2, 5-diphenyltetrazolium bromide (MTT) kit [18].

### Cell invasion and migration assays

Cell invasion and migration ability was performed by Transwell assay based on previously described [19].

### Western blot analysis

Cells were washed twice by pre-cooling phosphate buffered saline (PBS) and lysed for 30 min at 4 °C before centrifuged at 15,000×g for 15 min at 4 °C to remove cell debris. Then, the separated proteins were transferred onto the polyvinylidene fluoride (PVDF) membranes after using 10% sodium dodecyl sulfate polyacrylamide gel electrophoresis. To ensure all the samples were transferred, the PVDF membranes were stained with ponceau staining solution. Then, the membranes were incubated in sealing solution for 2 h at room temperature. Next, the membranes reacted with anti-Notch1 (1/500, ab8925, Abcam, Cambridge, MA, USA), anti-Notch2 (1/200, ab8926, Abcam) and anti-Notch3 (1 μg/mL, ab23426, Abcam) for 2 h. And then, the membranes were fully washed twice in PBS and twice in tris-buffered saline tween (TBST). Afterwards, the membranes were cultivated with secondary antibody goat anti-mice (1:1000, ab7068, Abcam) labeled by horseradish peroxidase (HRP) for 1 h, washed in TBST again and finally visualized with Super Signal West Pico kit. β-actin was applied as the internal reference.

### Fluorescence in situ hybridization (FISH) assay

MCF-7 and HCC1937 cells were hybridized with lncRNA SNHG3 probe (Exiqon, Vedbaek, Denmark). The probe mixture was denatured at 85 °C and the hybridization was stayed overnight at 65 °C. The sections were washed with sodium chloride-sodium citrate buffer with the original concentration. Then the slides were treated in 5% sealing solution for 30 min at room temperature, and each section was cultivated in sealing buffer overnight at 4 °C with the anti digoxigenin (NEF832001EA, Perkin-Elmer, Waltham, Massachusetts, USA) labeled by 100 μL HRP at the ratio of 1: 500. After 3 times of tris buffered saline (TBS) washes (10 min/time), trichostatin (TSA) staining solution was prepared in accordance with instructions of Perkin-Elmer TSA Plus kit (NEL753001KT, Perkin-Elmer). After that, the sections were incubated in TBS containing 4′, 6-diamidino-2-phenylindole (DAPI), washed and air-dried, and finally fixed in aqueous fluorescent mounting reagent. The pictures were captured using a Leica SP8 laser scanning confocal microscope (Leica, Solms, Germany).

### RNA pull-down assay

A total of 100 μg RNA was extracted. Then, 500 μg streptavidin beads were combined with miR-154 labeled with 200 pmol biotin, and incubated with the extracted RNA for 1 h. Next, the elution buffer was added to collect the pull-down RNA complex. The mRNA levels of lncRNA SNHG3 and Notch2 were quantitatively analyzed by RT-qPCR. The specific operations strictly followed the instructions of Magnetic RNA-Protein Pull-Down kit (GENEWIZ, Beijing, China).

### Dual luciferase reporter gene assay

Cells were transfected with 2 μg pMiR-report vector-SNHG3/Notch2 3′UTR (GenePharma, Shanghai, China) and miRNA-154-3p using Lipofectamine 2000. Transfected cells were lysed at 48 h and then luciferase activities were detected using Dual-luciferase Reporter Assay System. All the experiments were performed three times.

### Xenografts transplantation

Twelve specific pathogen-free BALB/c nude mice (4–6 week-old, 20 ± 2 g) [Beijing Vital River Laboratory Animal Technology Co., Ltd., Beijing, China, SCXK (Beijing) 2015–0001] were numbered with body weight as a parameter and randomly assigned into two groups (*n* = 6). The stably transfected 4 × 10^6^ MCF-7 cells by si-SNHG3 or Scramble siRNA were dispersed by 2 mL saline and injected subcutaneously into the right axilla of mice. Tumor volume was measured every 5 days and every 3 days after the 20th day. Mice were suffocated to death by CO_2_ 35 days later. The tumors were taken out and weighed for immunohistochemistry, with every step following the guidance in a literature report [20]. Primary antibodies used in the immunohistochemistry were anti-Notch1 (1/200, ab8925, Abcam), anti-Notch2 (1/200, ab8926, Abcam) and anti-Notch3 (5 μg/mL, ab23426, Abcam), as well as the secondary antibody (1:1000, ab150117, Abcam) labeled by HRP.

### Statistical analysis

All the experiments were performed in triplicate. The measurement data were expressed as mean ± standard deviation. Statistical analysis was performed with GraphPad Prism 8 software (GraphPad, San Diego, CA, USA). The *p*-values were calculated using the one-way or two-way analysis of variance (ANOVA). Tukey’s multiple comparisons test was used for the pairwise comparison after ANOVA analysis. An adjusted *p*-value < 0.05 was regarded as a statistically significant result.

## Results

### LncRNA SNHG3 was highly expressed in BC patients

**Fig. 1:**
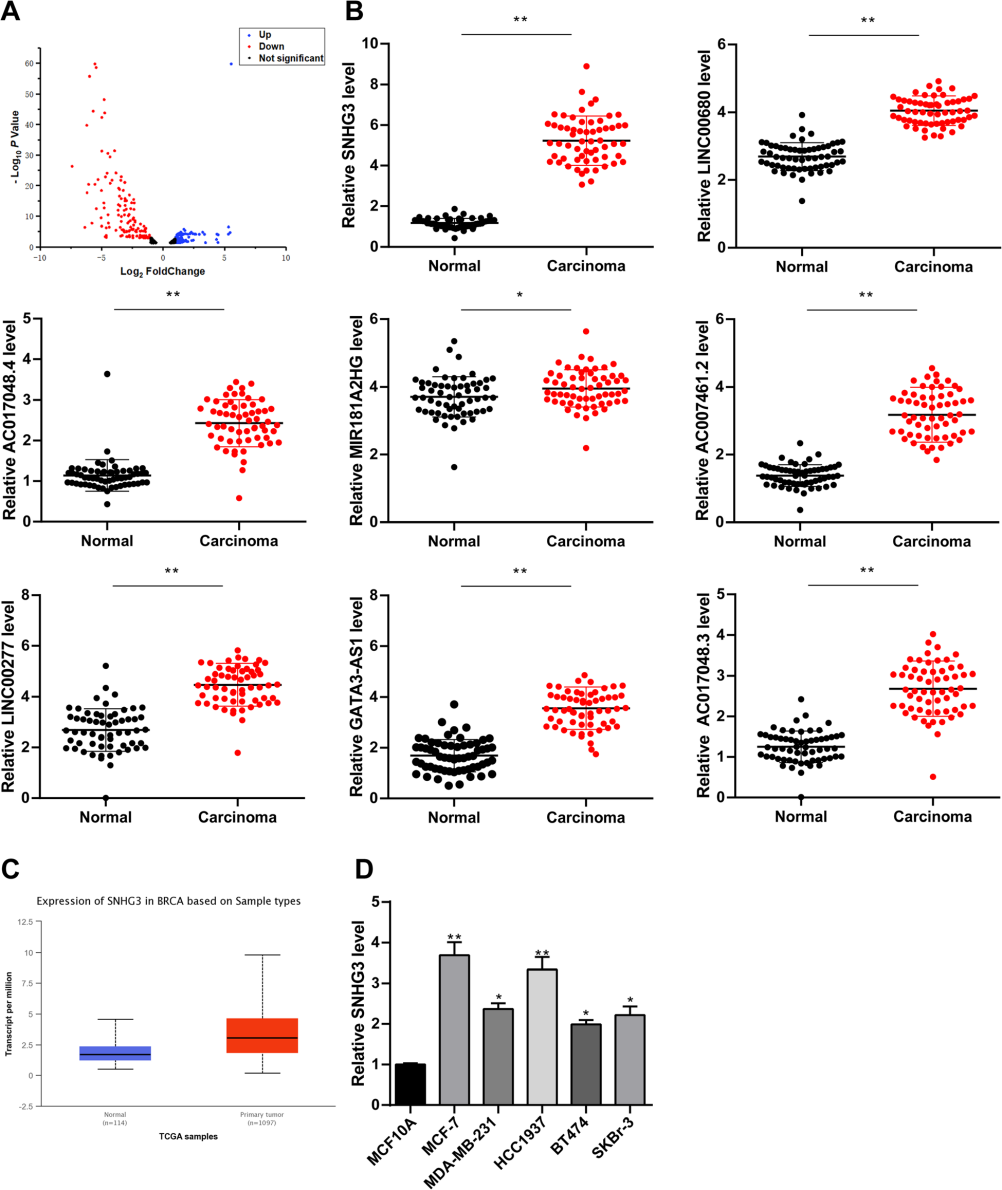
SNHG3 was upregulated in BC. **a**. Volcano map of lncRNAs between BC and benign breast lesions specimens by transcriptome analysis. The blue dots indicated high lncRNA expression; the red dots indicated low lncRNA expression and the black dots showed the lncRNAs with an expression of |log2FC| < 2. Log2FC was logarithm of fold-change with base 2 and the fold-change was cancer over normal. The Y axis represented an adjusted FDR, and the X axis represented the log2FC value. Aberrantly expressed lncRNAs were identified by DESeq R. Altogether, 137 highly expressed and 139 low expressed lncRNAs were identified; **b**. Different expressions of the top 8 lncRNAs between BC and benign breast lesions specimens by RT-qPCR; **c**. SNHG3 expression in normal tissue and primary tumor assessed by UALCAN; **d**. SNHG3 level among BC cell lines and human mammary epithelial cells detected using RT-qPCR. Three independent experiments were performed. Data are expressed as mean ± standard deviation; one-way ANOVA and Tukey’s multiple comparisons test was used, *p < 0.05, **p < 0.01

Firstly, the expression difference of lncRNA between BC tissues and normal breast tissues were detected by transcriptome sequencing. A total of 478 lncRNAs were obtained, 276 of which were differentially expressed, 137 of which were highly expressed, and 139 of which were poorly expressed in cancer tissues (Fig. 1a). Eight lncRNAs with the most significant differential expression were selected: SNHG3, LNC00680, AC017048.4, MIR181A2HG, AC007461.2, LNC00277, GATA3-AS1 and AC017048.3 (Table 2), and their levels were verified in 60 pairs of BC tissues and normal breast tissues. Result of RT-qPCR was consistent with that of transcriptome sequencing (*p* < 0.05) (Fig. 1b). Chen J. et al. have indicated in a literature report that lncRNA SNHG promoted osteosarcoma via sponging miR-196a-5p [21]. Liu L. et al. have suggested that lncRNA SNHG3 existed as an oncogene in lung adenocarcinoma, and upregulation of lncRNA SNHG3 promoted lung adenocarcinoma cell growth [22]. It has also been found that the malignancy of glioma was encouraged by SNHG3 via silent kruppel-like factor3 and p21 [23]. Taherian-Esfahani Z. et al. have found that lncRNA SNHG family played an important role in occurrence and hallmark of BC. SNHG1 expression was related to clinical staging; SNHG5 was related to malignance while SNHG3 expressed higher in estrogen receptor/progesterone receptor (ER/PR) compared with ER/PR positive BC [24]. However, there was less study about SNHG3 in BC. According to UALCAN (http://ualcan.path.uab.edu/index.html), an online bioinformatics analysis site [25], we found that lncRNA SNHG3 expression in BC patients was evidently higher than that in healthy people (*p* < 0.05) (Fig. 1c). Besides, SNHG3 had a higher expression in BC cell lines than that in MCF10A cells (*p* < 0.05) (Fig. 1d).

**Table 2:** Characteristics of the top 10 lncRNAs

Ensemble	Gene	Dysregulation	Fold Change	*P* Value
ENSG00000242125	SNHG3	Up	14.57442588	1.15E-68
ENSG00000215190	LNC00680	Up	3.616287083	3.75E-27
ENSG00000224577	AC017048.4	Up	7.63389477	5.67E-49
ENSG00000224020	miR-181A2HG	Up	10.62338023	1.55E-55
ENSG00000226101	AC007461.2	Up	4.326357933	5.34E-30
ENSG00000212766	LNC00277	Up	4.302321175	9.37E-47
ENSG00000197308	GATA3-AS1	Up	5.294820578	2.35E-41
ENSG00000163364	AC017048.3	Up	14.0691325	4.78E-24

Note: *SNHG3* Small nucleolar RNA host gene 3, *LNC* Long non-coding, *miR* microRNA

### Interfered lncRNA SNHG3 repressed BC cell proliferation, invasion and migration

**Fig. 2:**
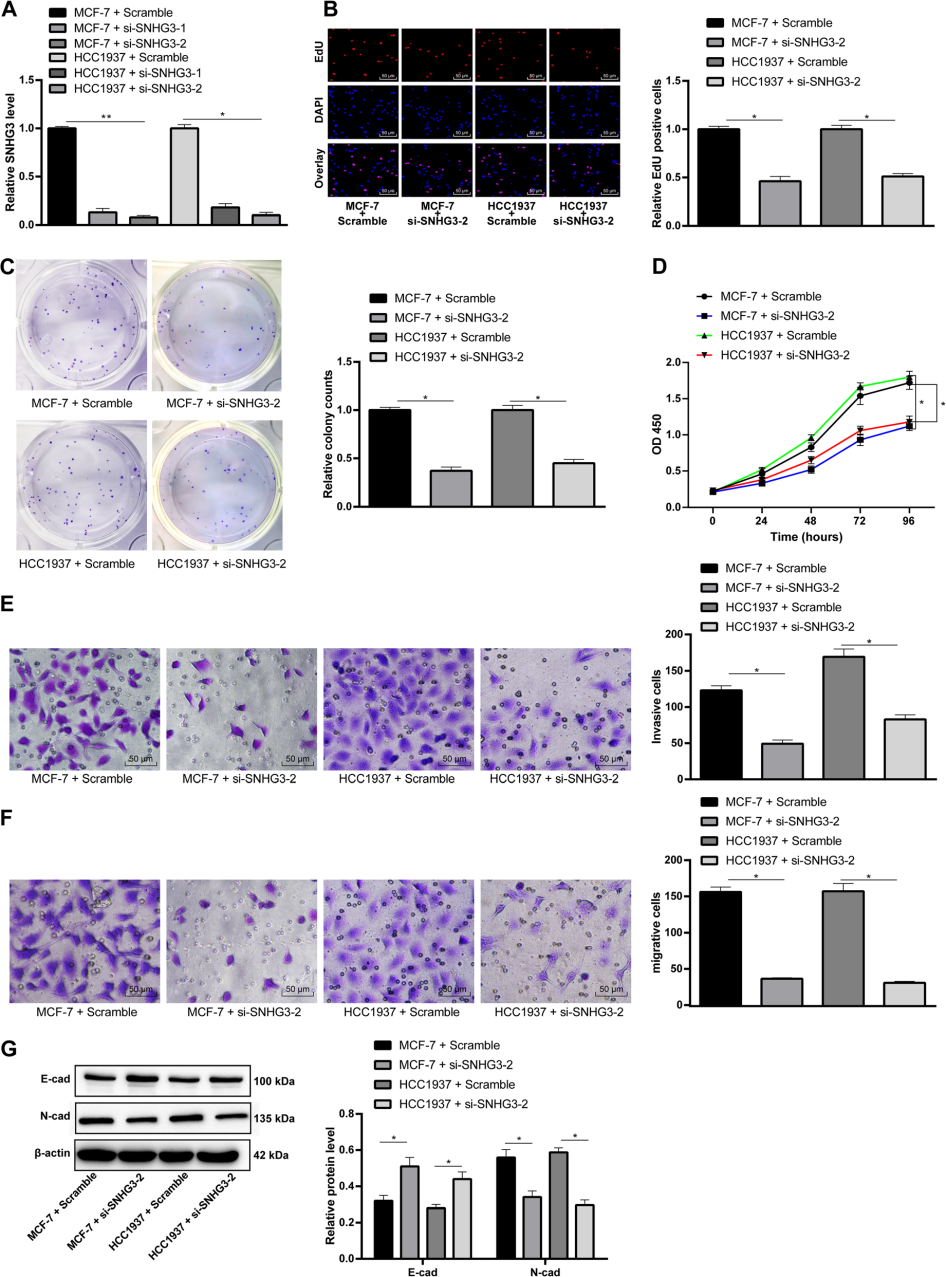
SNHG3 silencing effectively inhibited BC cells proliferation, invasion and migration. Two siRNAs targeted SNHG3 and scramble siRNA were transfected into MCF-7 and HCC1937 cells. **a**. RT-qPCR was performed to validate siRNA transfection. MCF-7 and HCC1937 cell biological behaviors were detected with EdU staining (**b**); BC cell proliferation detected by MTT proliferation assay (**c**) and colony formation assays (**d**); E. MCF-7 and HCC1937 cells migrating from upper Transwell chambers into lower ones, without Matrigel (× 200); **f**. MCF-7 and HCC1937 cells invading from Matrigel-coated upper Transwell chambers into lower ones (× 200); **g**. Western blot analysis was carried out to determine E-cadherin and N-cadherin protein levels (representative images were shown, full-length gels are presented in Supplementary Figure 1). Three independent experiments were performed. Data are expressed as mean ± standard deviation; one-way ANOVA and Sidak’s multiple comparisons test was used to determine statistical significance, or two-way ANOVA and Tukey’s multiple comparisons test was used, *p < 0.05, **p < 0.01

To further prove the effect of SNHG3 on BC cells, siRNA were used to construct MCF-7 and HCC1937 cells with stable knockdown of SNHG3. Firstly, after siRNA interference was verified by RT-qPCR, the expressions of SNHG3 in MCF-7 and HCC1937 cells showed an evident decline and siRNA-2 had a more powerful intervention capacity (*p* < 0.05) (Fig. 2a). Next, EdU staining, colony formation assay and MTT assay were performed to measure BC cell viability and proliferation. As the results shown, BC cell viability and proliferation significantly decreased after intervening SNHG3 (*p* < 0.05) (Fig. 2b-d). Invasion and migration of BC cells decreased obviously as showed by Transwell assay (*p* < 0.05) (Fig. 2e/f). The expressions of epithelial-mesenchymal transition (EMT)-related proteins E-cadherin (1:50, ab1416, Abcam) and N-cadherin (1:100, ab18203, Abcam) in BC cell were further tested by Western blot analysis. The result revealed that after the interference of SNHG3, the expression of E-cadherin increased remarkably while the expression of N-cadherin decreased (*p* < 0.05) (Fig. 2g).

### SNHG3 strengthened Notch2 viability by competitively combination with miR-154-3p

**Fig. 3:**
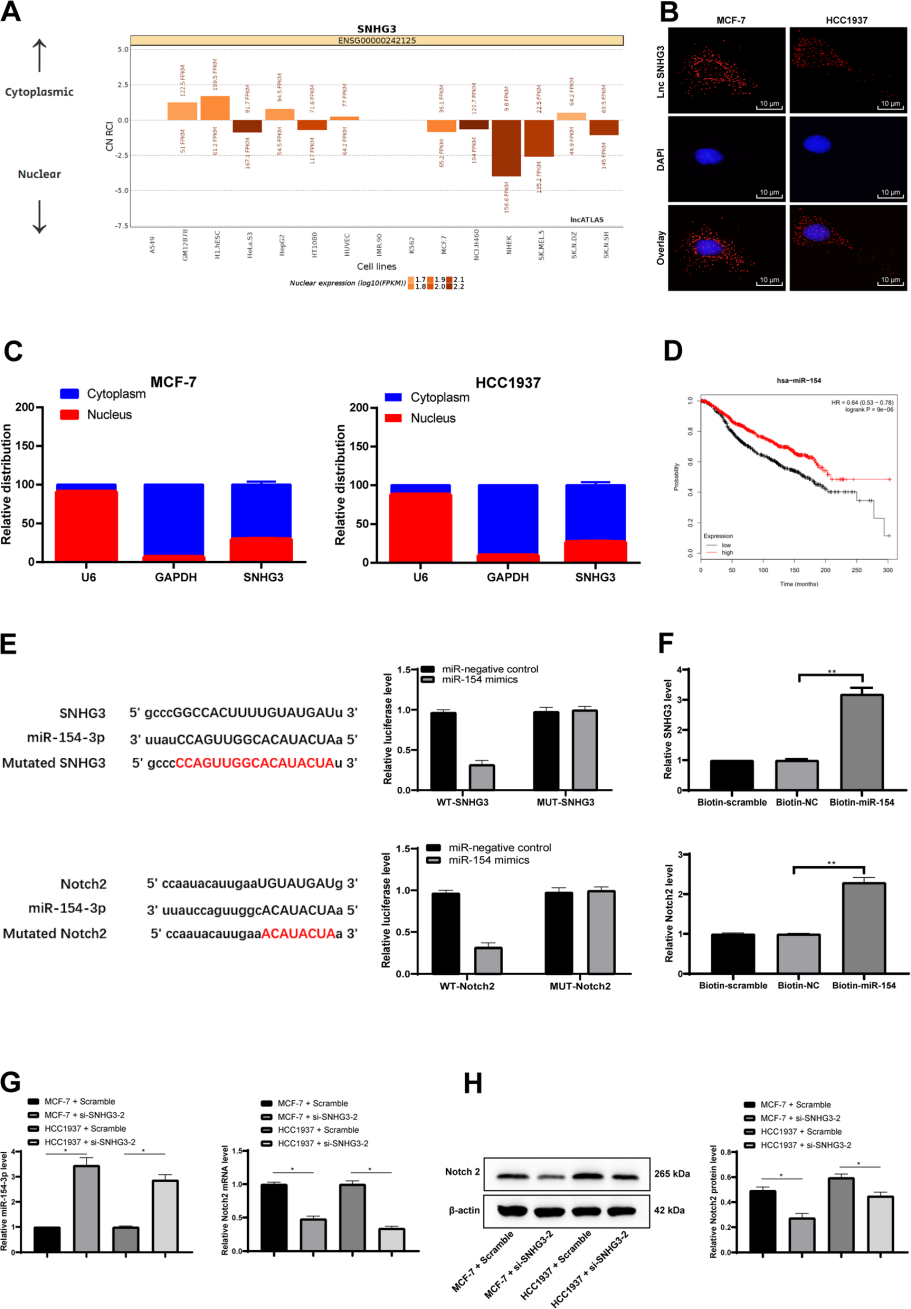
SNHG3 competitively bound to miR-154-3p and regulated Notch2. **a**. Subcellular localization of SNHG3 in the LncATLAS database; **b**. FISH experiments with probes targeting SNHG3 were performed to validate the subcellular localization of SNHG3 in MCF-7 and HCC1937 were stained with probes targeting SNHG3 (red stain), and the nuclei were stained with 4′,6-diamidino-2-phenylindole (blue stain). The merged image showed SNHG3 was cytoplasm-sublocalized in MCF-7 and HCC1937; **c**. Nuclear and cytoplasmic expression of SNHG3 in MCF-7 and HCC1937 cells determined by RT-qPCR; **d**. Kalpan-Meier plotter predicted breast cancer prognosis via miR-154-3p expression level; **e**. Luciferase reporter plasmid containing SNHG3-WT or SNHG3-Mut was transfected into 293 T cells together with miR-154-3p in parallel with an miR-NC plasmid vector; luciferase reporter plasmid containing SNHG3-WT or SNHG3-Mut was transfected into 293 T cells together with miR-154-3p in parallel with an miR-NC plasmid vector; luciferase reporter plasmid containing NOTCH2-WT or NOTCH2-Mut was transfected into 293 T cells together with miR-154-3p in parallel with an miR-NC plasmid vector; **f**. the binding relationship between miR-154-3p, SNHG3 and Notch2 was verified by RNA pull-down assay; **g**. RT-qPCR was performed to determine the levels of miR-154-3p and Notch2 mRNA in MCF-7 and HCC1937 cells.; **h**. Western blot assay was performed to determine Notch2 protein level in MCF-7 and HCC1937 cells (representative images were shown, full-length gels are presented in Supplementary Figure 2); J. RT-qPCR and western blot analysis were performed to determine Notch2 level in MCF-7 and HCC1937 cells. Three independent experiments were performed. Data are expressed as mean ± standard deviation; one-way ANOVA and Tukey’s Multiple comparison test were used to determine statistical significance, *p < 0.05

Firstly, lncATLAS database (http://lncatlas.crg.eu/) [26] was used to predict that the subcellular fractions of lncRNA SNHG3 were mainly localized in cytoplasm (Fig. 3a). Afterwards, FISH assay verified that lncRNA SNHG3 was mainly localized in the cytoplasm of MCF-7 and HCC1937 cells. The probes of lncRNA SNHG3 in MCF-7 and HCC1937 cells were stained into red, and the nucleus was stained into blue by DAPI (Fig. 3b). Then, the total RNA of MCF-7 and SNHG3 cells was extracted by separating cytoplasm and nucleus to detect lncRNA SNHG3 expression in cytoplasm and nucleus respectively. As showed in Fig. 3c, SNHG3 mainly appeared in cytoplasm (*p* < 0.05), suggesting that SNHG3 affected the development of BC through the mechanism of CeRNA. Thereafter, a large number of miRs were predicted to be possibly combined with SNHG3 by Starbase (http://starbase.sysu.edu.cn/) [27], and we focused on miR-154-3p, which was regarded as a tumor suppressor in bladder cancer by targeting ATG7 according to Junfeng Wang et al. [28]. According to Hui Hu et al., BC cell proliferation and migration were inhibited when miR-154 targeted E2F5 transcription factors [29]. Kalpan-Meier plotter (http://kmplot.com/analysis/index.php? P = Service) [30] website was emplyed to predict the relationship between miR-154 and prognosis of BC patients, and it was found that patients with low expression of miR-154 had worse prognosis (Fig. 3d). In addition, dual luciferase reporter gene assay was conducted to verify the binding relation between miR-154-3p and SNHG3; the result of RNA pull-down experiment also revealed that there was a binding complex between SNHG3 and miR-154-3p; specifically, SNHG3 could be detected in the bio-miR-154 group, (*p* < 0.05) (Fig. 3e/f). Then, RT-qPCR was applied to verify the miR-154-3p expression in MCF-7 and HCC1937 cells after intervening SNHG3 expression. As showed in Fig. 3g, miR-154-3p expression was evidently increased after the intervention of SNHG3 (*p* < 0.05). Later, we further considered the downstream mechanism of miR-154-3p and predicted the target gene of miR-154 on Starbase website. And we focused on Notch2 by consulting the literature. Anuradha Sehrawat et al. have found that activating Notch discouraged BC cell apoptosis at initial stage [31]. The dual luciferase reporter gene assay confirmed the binding relation between miR-154 and Notch2, and RNA pull-down assay verified that miR-154 and Notch2 colud form binding complex (*p* < 0.05) (Fig. 3e/f). After that, RT-qPCR and Western blot analysis were employed to detect Notch2 expression in MCF-7 and HCC1937 cells after intervening SNHG3 expression. The expression of Notch2 was obviously decreased after intervention of SNHG3 (*p* < 0.05) (Fig. 3h). From the above results, it was concluded that SNHG3 enhanced Notch2 activity by competitively binding to miR-154-3p, thus promoting BC cell proliferation and metastasis.

### Activation of the notch signaling pathway partly reversed the inhibition of cell activity induced by intervening SNHG3

**Fig. 4:**
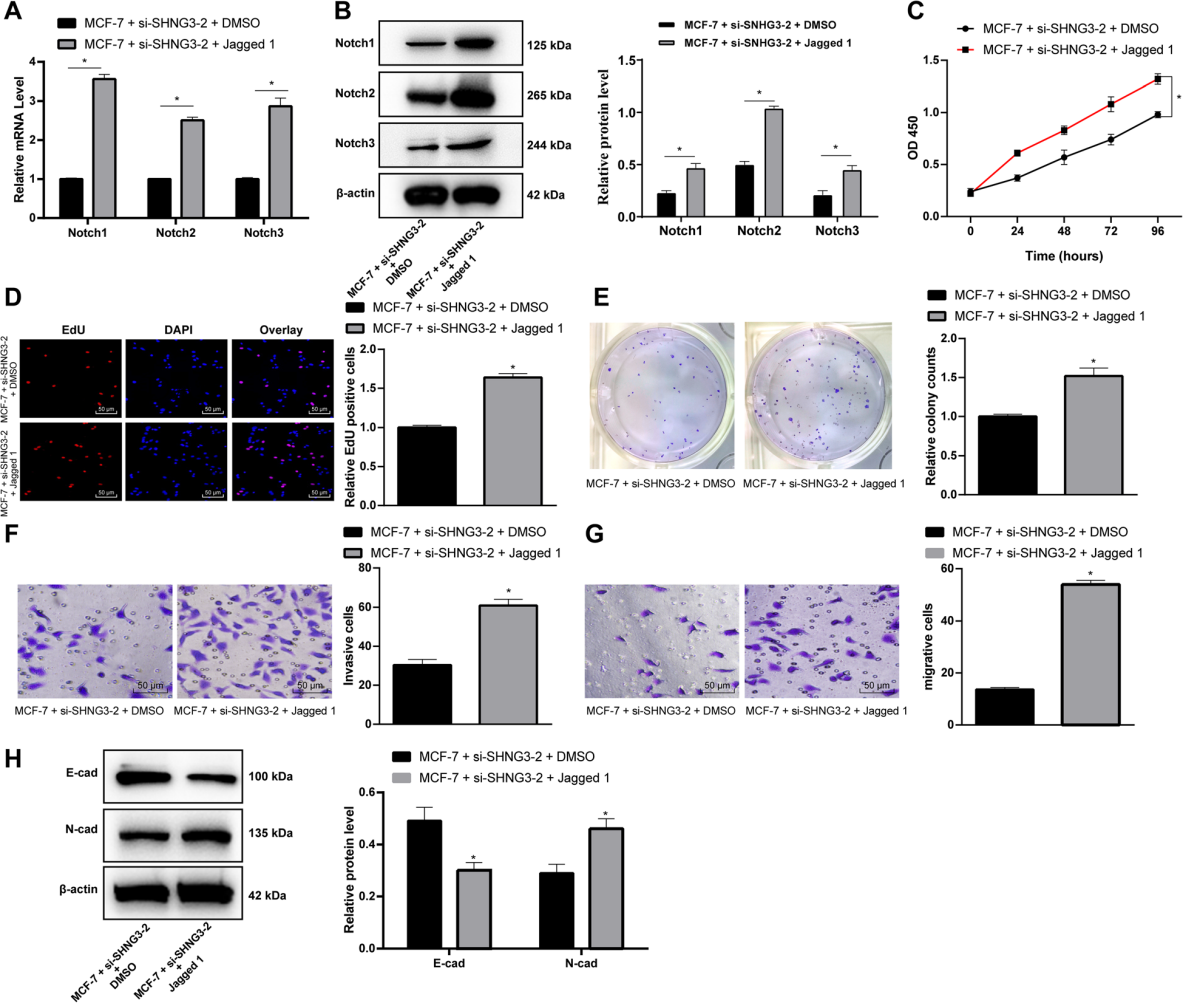
Notch signaling pathway activation reversed BC cells proliferation and viability by SNHG3 silencing. MCF-7 stably expressed si-SNHG3–2 was treated with Notch signaling pathway specific activator, Jagged 1. RT-qPCR and Western blot analysis were performed to determine Notch1, Notch2 and Notch3 mRNA (**a**) and protein (**b**) levels after Jagged 1 treatment (representative images were shown, full-length gels are presented in Supplementary Figure 3); MCF-7 cells were performed with MTT proliferation assay (**c**) and EdU staining (**d**) and colony formation assays (**e**) to determine Notch signaling pathway activation effectiveness; **f**. MCF-7 cells migrating from upper Transwell chambers into lower ones, without Matrigel (× 200); **g**. MCF-7 cells invading from Matrigel-coated upper Transwell chambers into lower ones (× 200); **h**. Western blot analysis was carried out to determine E-cadherin and N-cadherin protein level (representative images were shown, full-length gels are presented in Supplementary Figure 4). Three independent experiments were performed. Data are expressed as mean ± standard deviation; one-way ANOVA and Sidak’s multiple comparisons test was used to determine statistical significance, or two-way ANOVA and Tukey’s multiple comparisons test was used, *p < 0.05, **p < 0.01

Jagged 1, a specific activator of the Notch signaling pathway, was added into MCF-7 cells after intervening SNHG3 expression. The results of RT-qPCR and Western blot analysis showed that mRNA and protein levels of Notch1, Notch2 and Notch3 improved apparently (*p* < 0.05) (Fig. 4a/b), accompanied by the improvement of cell activity, proliferation, invasion and migration (*p* < 0.05) (Fig. 4c-h).

### SNHG3 intervention inhibits the growth of BC cell xenograft tumor in vivo

**Fig. 5:**
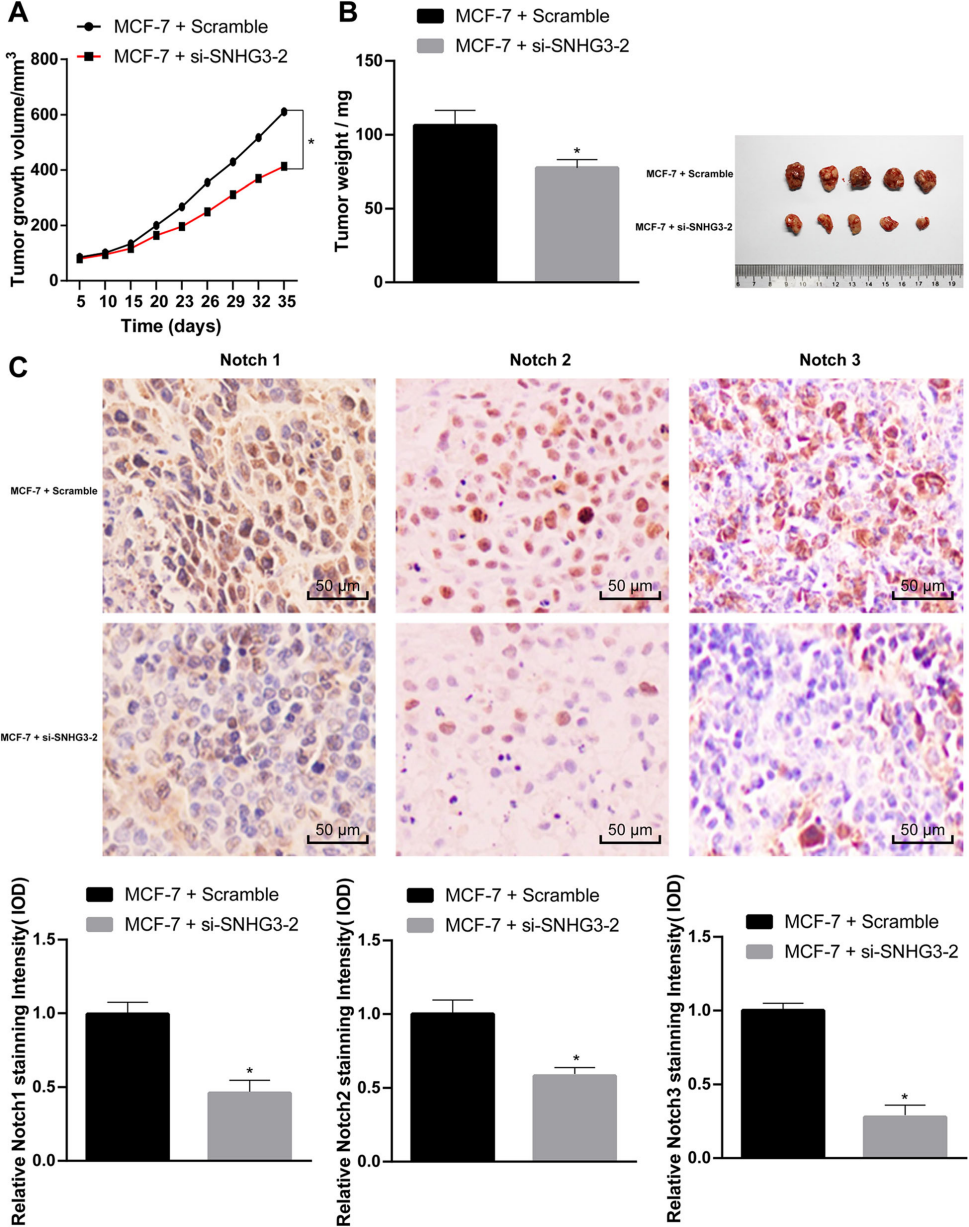
SNHG3 intervention can inhibit the growth of BC cell xenograft tumor in vivo. MCF-7 cells stably SNHG3-siRNA and scramble siRNA were inoculated subcutaneously into BALB/c nude mice at a dose of 5 × 10^6^ per mouse (*n* = 6 in each group). Tumor growth was measured continuously every 5 days, and 20 days later, tumor growth was monitored every 3 days. At 35 days post-implantation, the mice were euthanized by carbon dioxide asphyxiation. **a**. Tumor size; **b**. Tumor weight and representative view of xenografts. Tumor sections were obtained and stained with anti-Notch1, anti-Notch2 and anti-Notch3 antibodies; **c**. Representative views of Notch1, Notch2 and Notch3-positive tumor cells and quantification of immunohistochemistry. Data are expressed as mean ± standard deviation. One-way ANOVA and Sidak’s multiple comparisons test was used to determine statistical significance, or two-way ANOVA and Tukey’s multiple comparisons test was used, *p < 0.05, **p < 0.01

The growth and weight of transplanted tumors were measured to evaluate the effect of SNHG3 on MCF-7 cells in vivo. It was showed that inhibited SNHG3 suppressed the growth of tumor (*p* < 0.05) (Fig. 5a/b). The result of immunohistochemistry revealed that after the inhibition of SNHG3 expression, Notch1-, Notch2- and Notch3-positive cells in MCF-7 xenograft tumor increased (*p* < 0.05) (Fig. 5c).

## Discussion

As the most common malignant cancer and main cause of mortality in women, BC showed a high survival rate, but reducing BC incidence and mortality remains a priority for the public [32]. Besides, lncRNAs are deregulated in a variety of cancers and regulate cancer-related pathways, indicating that they play vital roles in cancer prognosis [33]. A prior study has demonstrated that lncRNA MIAT promotes BC progression and functions as ceRNA to regulate DUSP7 expression by sponging miR-155-5p [34]. In this study, we assumed that there may be roles of lncRNA SNHG3 in BC cell proliferation and metastasis via the Notch signaling pathway. Consequently, our data showed that SNHG3 competitively bound to miR-154-3p and activated the Notch signaling pathway to promote BC cell proliferation and metastasis.

Firstly, the results of transcriptome sequencing showed that SNHG3 was expressed higher in BC cells than that in normal breast cells. Consistently, another study reported that SNHG3 expression was remarkably higher in ovarian cancer tissues than in adjacent normal tissues, and upregulating SNHG3 expression linked with poor prognosis and enhanced malignant progression of ovarian cancer [35]. LncRNA SNHG3 was proved to be upregulated in BC cells [7]. Functional assays by Liang Liu et al. have suggested that upregulated SNHG3 led to growth of cell proliferation, cell cycle progress and decrease of cell apoptosis, indicating that SNHG3 served as an oncogene in lung cancer by controlling tRNA processing, transcription, apoptosis, cell adhesion and signal transduction [22]. Additionally, this current study also suggested that BC cell proliferation, invasion and migration evidently decreased with inhibited SNHG3. Lan Hong and his colleagues found that ovarian cancer cell proliferation and invasion were inhibited after SNHG3 knockdown [35]. Similarly, SNHG1 promoted miR-448 expression, suppressed regulatory T cell differentiation, and eventually impeded the immune escape of BC [36]. Meanwhile, a recent article has indicated that overexpressed SNHG3 encouraged osteosarcoma (OS) cell invasion and migration, lessening the survival rate of OS patients [37]. That’s to say, a higher survival rate could be achieved by the inhibition of SNHG3. Therefore, poor expression of SNHG3 might act as a possible therapeutic target for BC. What’s more, functional assays in our study found that the E-cadherin level was expressly enhanced and N-cadherin level was noticeably declined after interfering SNHG3. As a tumor suppressor, E-cadherin played an important role in encouraging BC cell progression and metastasis [38]. N-cadherin expression promoted BC cell mobility, invasion and migration [39]. So, interfered SNHG3 could repress BC cell biological behaviors.

Additionally, dual-luciferase reporter gene assay found a link between SNHG3 and miR-154-3p. Then, we focused on miR-154-3p. Recently, it has been found that in BC cells where lncRNA SNHG5 was negatively correlated with miR-154-5p, increase of SNHG5 suppressed miR-154-5p and upregulated proliferation cell nuclear antigen, promoting BC cell biological processes [40]. Another study has unearthed that SNHG1 served as a sponge in weakening miR-154-5p, which could regulate BC cell proliferation and apoptosis [41]. Besides, in our study, the binding relation between miR-154-3p and Notch2 was also found in a dual-luciferase reporter gene assay. Highly expressed Notch2 was found to improve survival rate in many BC patients and was important in Notch signaling pathway activation [42, 43]. Mattia Capulli et al. have demonstrated that BC cell proliferation was repressed by endosteal niche cells in a Notch2-related way [12]. However, this study was the first to explore the molecular mechanism between SNHG3, miR-154 and Notch2 pathway. It was found that SNHG3 could act as a ceRNA of miR-154-3p and upregulate the Notch signaling pathway to promote BC cell proliferation and metastasis. Moreover, RT-qPCR and Western blot analysis found that activating the Notch signaling pathway encouraged BC cell viability, proliferation, invasion and migration. Previous research suggested that aberrant Notch signaling pathway played a significant role in implicating BC cell progression [44], which was in agreement with our results. Interestingly, effect of Notch2 on BC cell proapoptotic and anti-migratory response has been revealed to be inhibited when it was activated by zerumbone [31]. Furthermore, Notch signaling pathway has been found to play a significant role in breast epithelial cell differentiation and participated in BC growth by Notch receptors and ligands [45].

## Conclusion

In summary, our study supported that lncRNA SNHG3 promoted BC cell proliferation and metastasis by competitively binding to miR-154-3p and activating the Notch signaling pathway. Now, molecule-targeted treatment of tumors has been widely accepted. The results in this study may provide novel insights for the molecular therapy of BC. In the future, we will further explore the mechanism of other targets of lncRNA SNHG3, and explore the role of Notch 1 and Notch 3 in breast cancer. We will carry out relevant researches, for example the rescue experiments in which inhibition of miR-154 negates growth inhibitory effects caused by SNHG3 knockdown under the permission of experimental conditions and funds. Although our findings provide therapeutic implication in BC treatment, the experiment results and effective application into clinical practice need further validation.

## Supplementary information

**Additional file 1.**

**Additional file 2.**

**Additional file 3.**

**Additional file 4.**

## Data Availability

The datasets used and/or analysed during the current study available from the corresponding author on reasonable request.

## Abbreviations

ANOVA: Analysis of variance
BC: Breast cancer
ceRNA: Competing endogenous RNA
DAPI: 4′,6-diamidino-2-phenylindole
EdU: 5-ethynyl-2′-deoxyuridine
EMT: Epithelial-mesenchymal transition
ER/PR: Estrogen receptor/progesterone receptor
FISH: Fluorescence in situ hybridization
HRP: Horseradish peroxidase
lncRNA: Long non-coding RNA
miR: MicroRNA
MTT: 3-(4, 5-dimethylthiazol-2-yl)-2, 5-diphenyltetrazolium bromide
OC: Ovarian cancer
OS: Osteosarcoma
PBS: Phosphate buffered saline
PVDF: Polyvinylidene fluoride
RT-qPCR: Reverse transcription quantitative polymerase chain reaction
siRNA: Small interfere RNA
SNHG3: Small nucleolar RNA host gene 3
TBS: Tris buffered saline
TBST: Tris-buffered saline tween
TSA: Trichostatin
