# Supplementary figures and images for: Long non-coding RNA SNHG3 promotes breast cancer cell proliferation and metastasis by binding to microRNA-154-3p and activating the notch signaling pathway
Source: BMC Cancer. 2020 Sep 3;20:838. doi: 10.1186/s12885-020-07275-5 (PMC7469338; doi:10.1186/s12885-020-07275-5)

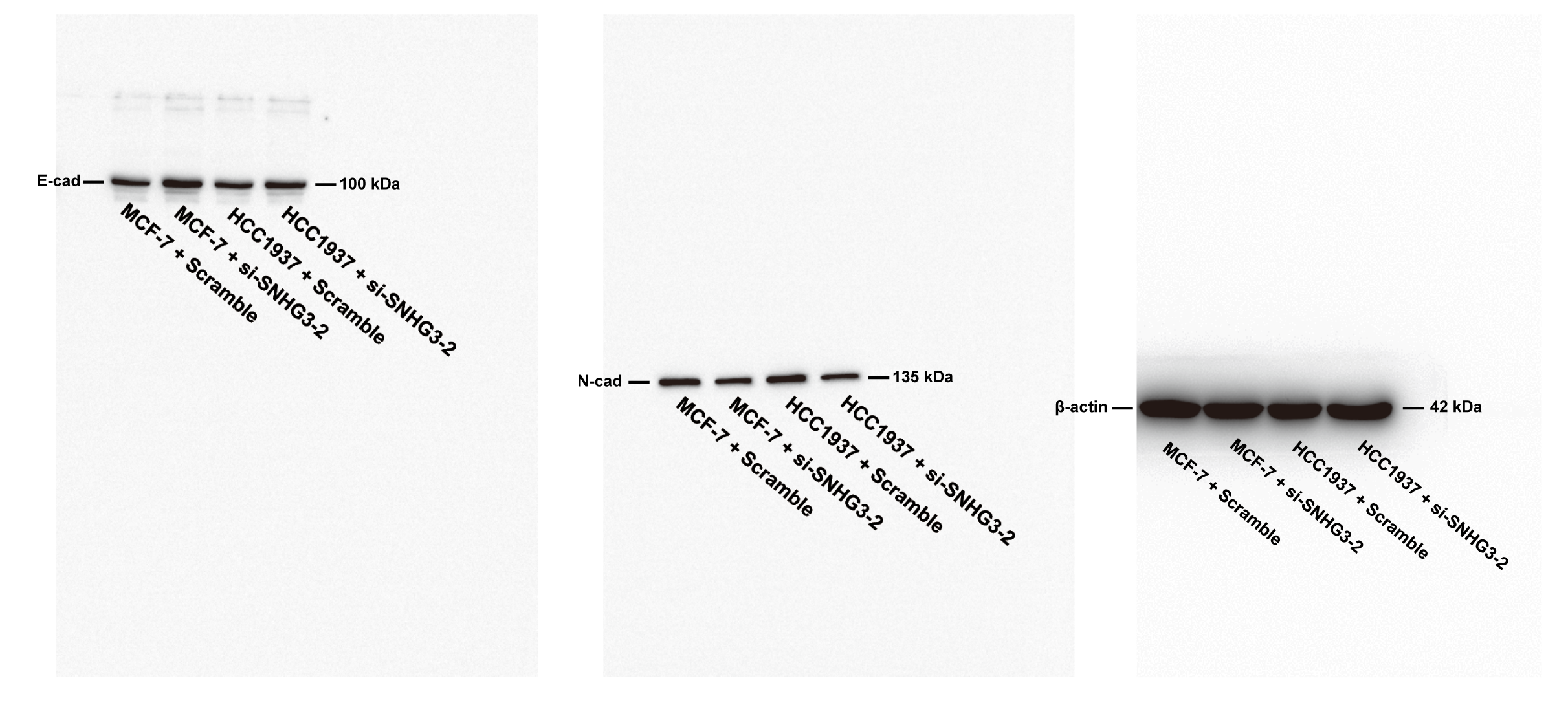

Supplement: Supplementary file 1 — Additional file 1. [file 12885_2020_7275_MOESM1_ESM.tiff]

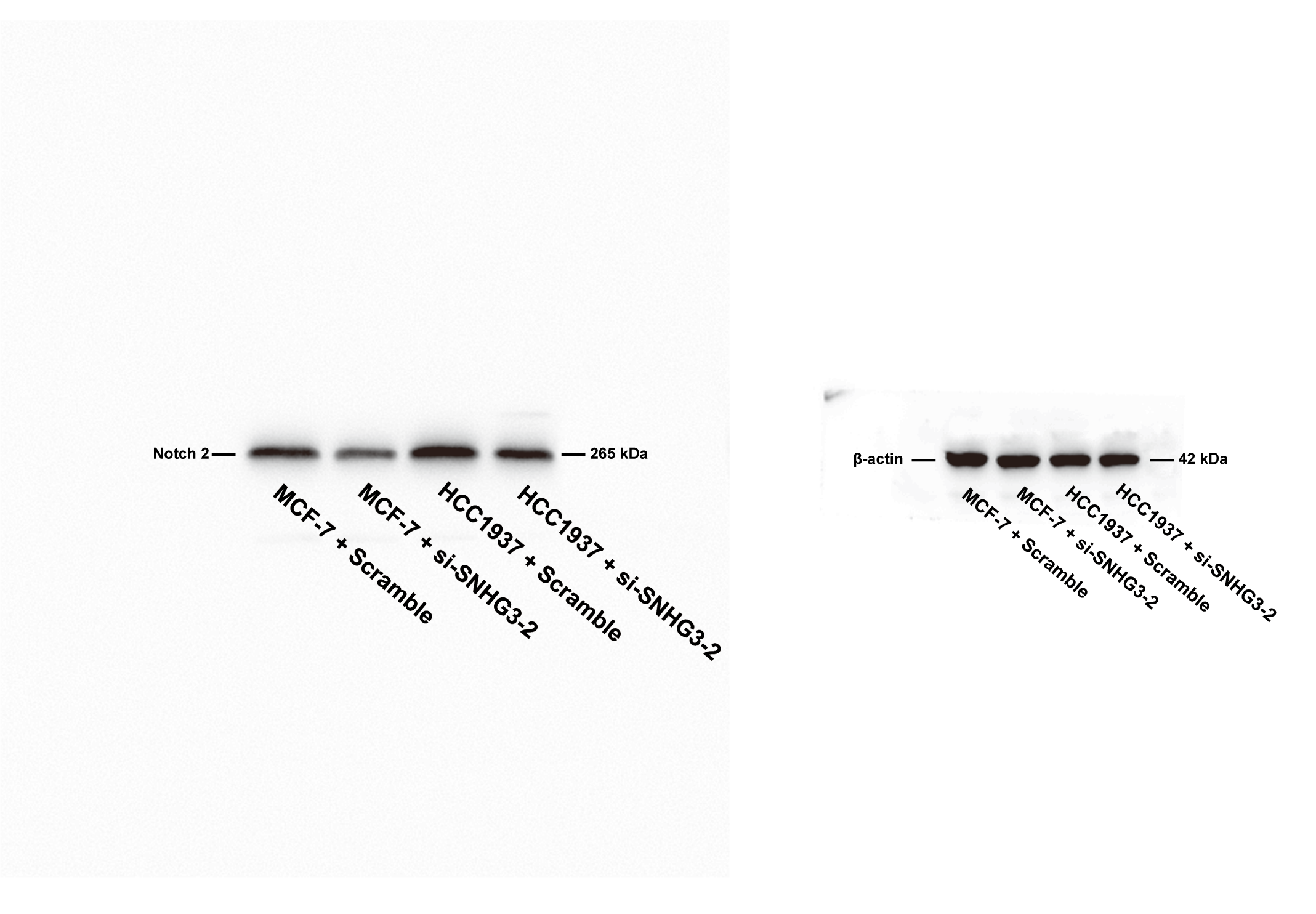

Supplement: Supplementary file 2 — Additional file 2. [file 12885_2020_7275_MOESM2_ESM.tiff]

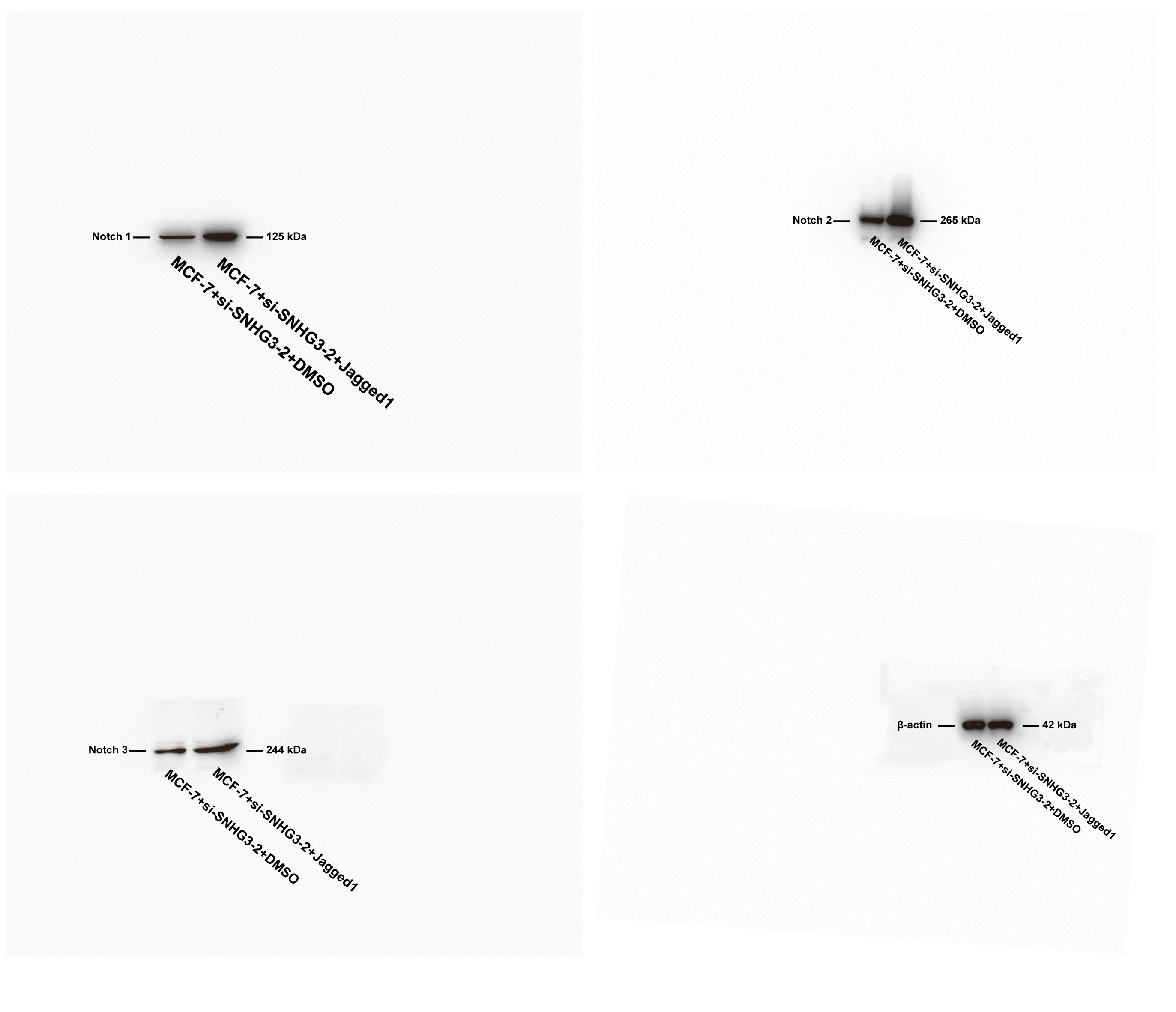

Supplement: Supplementary file 3 — Additional file 3. [file 12885_2020_7275_MOESM3_ESM.tiff]

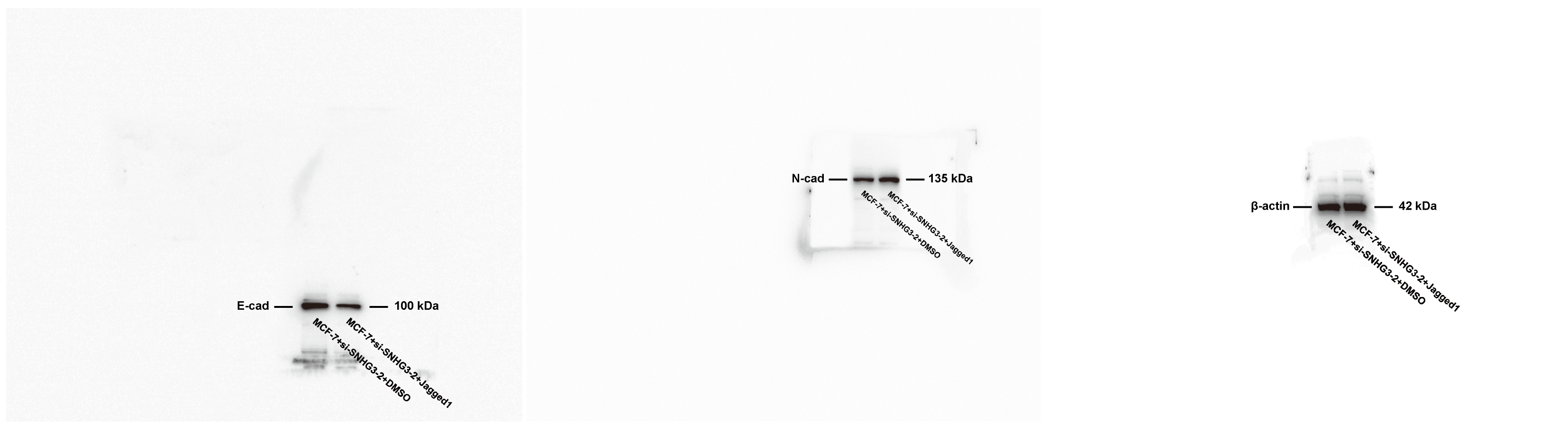

Supplement: Supplementary file 4 — Additional file 4. [file 12885_2020_7275_MOESM4_ESM.tiff]
